# A Pragmatic Approach to Susceptibility Classification of Yeasts without EUCAST Clinical Breakpoints

**DOI:** 10.3390/jof8020141

**Published:** 2022-01-30

**Authors:** Karen Marie Thyssen Astvad, Sevtap Arikan-Akdagli, Maiken Cavling Arendrup

**Affiliations:** 1 Unit of Mycology, Statens Serum Institut, DK-2300 Copenhagen, Denmark; kaas@ssi.dk; 2 Department of Medical Microbiology, Hacettepe University Medical School, Ankara 06100, Turkey; sarikan@hacettepe.edu.tr; 3 Department of Clinical Microbiology, Rigshospitalet, DK-2100 Copenhagen, Denmark; 4 Department of Clinical Medicine, University of Copenhagen, DK-1165 Copenhagen, Denmark

**Keywords:** rare yeast, antifungal susceptibility testing, EUCAST, clinical breakpoint, epidemiological cut-off value, ECOFF

## Abstract

EUCAST has established clinical breakpoints for the six most common *Candida* species and *Cryptococcus neoformans* but not for less common yeasts because sufficient evidence is lacking. Consequently, the question “How to interpret the MIC?” for other yeasts often arises. We propose a pragmatic classification for amphotericin B, anidulafungin, fluconazole, and voriconazole MICs against 30 different rare yeasts. This classification takes advantage of MIC data for more than 4000 isolates generated in the EUCAST Development Laboratory for Fungi validated by alignment to published EUCAST MIC data. The classification relies on the following two important assumptions: first, that when isolates are genetically related, pathogenicity and intrinsic susceptibility patterns may be similar; and second, that even if species are not phylogenetically related, the rare yeasts will likely respond to therapy, provided the MIC is comparable to that against wild-type isolates of more prevalent susceptible species because rare yeasts are most likely “rare” due to a lower pathogenicity. In addition, the treatment recommendations available in the current guidelines based on the in vivo efficacy data and clinical experience are taken into consideration. Needless to say, it is of utmost importance (a) to ascertain that the species identification is correct (using MALDI-TOF or sequencing), and (b) to re-test the isolate once or twice to confirm that the MIC is representative for the isolate (because of the inherent variability in MIC determinations). We hope this pragmatic guidance is helpful until evidence-based EUCAST breakpoints can be formally established.

## 1. Introduction

The goal of in vitro antifungal susceptibility testing (AFST) according to the EUCAST (European Committee on Antimicrobial Susceptibility Testing) method is to inform the clinicians whether an antifungal drug is appropriate for an infection caused by a specific fungal isolate [1]. EUCAST clinical breakpoints allow classification of the organism as “susceptible” or “resistant” to standard dosing. Furthermore, EUCAST has a third category “susceptible, Increased exposure” indicating that the organism is susceptible if higher than standard exposure is achieved, either by increasing the dose or if the compound is concentrated at the site of infection [1,2]. EUCAST breakpoints are established taking the following available parameters into consideration: (a) dosing regimens used; (b) MIC distributions from multiple laboratories; (c) species- and compound-specific epidemiological cut-off values (ECOFFs); (d) pharmacokinetic/pharmacodynamic relationships and targets associated with outcome, and finally, (e) clinical outcome data by species and MIC [3].

Unfortunately, many fungal species still lack breakpoints for some or all antifungal agents [1]. This particularly applies to the uncommon or rare *Candida* species and other yeasts, which, although being reported as causative agents of invasive infections in various multi-centre or nationwide studies, are indeed many fold less common [4,5,6,7,8,9,10,11,12] (Table 1). The lack of breakpoints complicates the interpretation of antifungal susceptibility test results and consequently the optimal choice of therapy. ESCMID and ECMM joint clinical guidelines were issued in 2014 for diagnosis and treatment of rare invasive yeast infections [13] and were recently revised as the global guideline as an initiative of ECMM in cooperation with ISHAM and ASM [14]. These guidelines do not include breakpoints for interpretation of individual MICs but do describe the level of evidence of clinical efficacy and recommended treatment.

Here, we discuss basic concepts of susceptibility testing and associated terminology. Furthermore, we discuss how to proceed when faced with an MIC against a yeast without breakpoint. Finally, we comment on various species without breakpoints and provide a pragmatic approach to interpretation of antifungal susceptibility test results for amphotericin B, anidulafungin, fluconazole, and voriconazole.

Of note, fungal taxonomy is under constant and considerable revision. While many genera have both teleomorphic (sexual state) and anamorphic (asexual state) names, the “One Fungus One Name” initiative strives to determine one current name for each species. Furthermore, DNA-based phylogeny studies have demonstrated cryptic species that were previously morphologically or phenotypically indistinguishable from parent species, and genera and species name changes are abundant and ongoing [15,16]. In this article, we use mainly the traditional anamorph name (current name) in concordance with EUCAST documents. A list of current and previous yeast species names used in this document is available in Supplementary List S1.

## 2. What Is in an MIC?

Susceptibility data describe how a given isolate, grown under standardised conditions, responds to increasing concentrations of antifungal agents in the laboratory. The lowest concentration of the drug (mg/L) needed to achieve a given endpoint of suppressed growth is reported as an MIC (minimal inhibitory concentration). The growth is influenced by the media, nutrient concentrations, inoculum size, temperature, incubation time, endpoint definition (often in relation to an antifungal free growth control), microplate type, and, finally, antifungal concentrations. Historically, national reference methods have been developed and proposed, but these are now virtually all replaced by the methodologies and breakpoints developed and documented by EUCAST in Europe and CLSI in the USA [1,2,17,18,19,20]. Both the EUCAST and CLSI reference methods for testing yeasts are broth microdilution methods, and the methods are more alike than different. Yet the following differences between EUCAST E.Def 7.3.2 and CLSI M27 4th ed. are worth noticing: a 10-fold higher glucose concentration, a 100-fold higher yeast conidia inoculum, flat-bottom wells and spectrophotometric endpoint reading for EUCAST compared to round-bottom plates and visual endpoint reading for CLSI [2,18]. Although harmonisation efforts have been undertaken (including reducing the reading time for CLSI MICs from 48 to 24 h and introducing species-specific breakpoints), MICs obtained by the different methods still differ for a number of agents and species and so do the corresponding breakpoints [21,22,23,24].

Clinical breakpoints are dependent on reliable species identification of the isolates. This is particularly true when MICs are interpreted in clinical practice based on single determinations, given the inherent variation in the test. Cryptic species may have inherently different susceptibility than the parent species, which may or may not have clinical relevance. Examples are *C. orthopsilosis* and *C. metapsilosis,* which have previously not always been differentiated from *C. parapsilosis* sensu stricto. One other example is *Cryptococcus gattii,* which has now been differentiated from *C. neoformans-gattii* species complex [12,25,26,27]. Clinical breakpoints are revised as needed when new or more information on MIC distributions, target mutations, PK-PD and clinical outcome becomes available.

**Table 1:** Overview of yeast species distribution among invasive infections based on selected studies.

Country/Region, Type of the Study [Reference]	Asian Multi-centre (25 Hospitals) [4]	Spain, Multi-centre (29 Centres) [5]	Sweden, Nationwide [6]	Italy, Lombardy Multi-centre (12 Hospitals) [7]	Denmark Nationwide [8,9]	Greece Single Centre (Tertiary Hospital) [10]	Norway Nationwide [11]	SENTRY 39 Countries [12]
Period (year)	2010–2011	2010–2011	2015–2016	2016–2017	2012–2018	2009–2018	2004–2012	2006–2016
Infection type	Blood/bone marrow	Bloodstream	Bloodstream	Bloodstream	Bloodstream	Bloodstream	Bloodstream	Bloodstream/Invasive
Main identification procedures ^1^	Variable methods Molecular ID (four)	ITS sequencing (all isolates)	>96% identified also by MALDI-TOF MS or VITEK MS	VITEK 2 (one), MALDI-TOF-MS, (two), VITEK MS (nine)	MALDI-TOF, ITS sequencing	VITEK 2 and Auxacolor	VITEK 2 and API 32. MALDI-TOF since 2011 Molecular ID	Sequence-based or proteomic methods
**Yeasts** isolates, *n*	2155	781	487	1020	3379	477	1724	15,312
*Candida*, *n* (%) ^2^	1988 (92.3)	766 (98.1)	485 (99.6)	1006 (98.6)	3333 (98.6)	449 (94.1)	1724	15,312
* C. albicans*		348 (44.6)	267 (54.8)	547 (53.6)	1540 (45.6)	186 (39.0)	1168 (67.7)	7179 (46.9)
* C. glabrata* SC ^3^		103 (13.2)	96 (19.7)	205 (20.1)	1084 (32.1)	48 (10.1)	255 (14.8)	2860 (18.7)
* C. parapsilosis* SC ^3^		191 (24.5)	44 (9.0)	161 (15.8)	126(3.7)	167 (35.0)	74 (4.3)	2433 (15.9)
* C. tropicalis*		59 (7.6)	18 (3.7)	56 (5.5)	158 (4.7)	31 (6.5)	112 (6.7)	1418 (9.3)
* C. krusei*		15 (1.9)	14 (2.9)	10 (1.0)	148 (4.4)	5 (1.0)	23 (1.3)	421 (2.7)
**Rare *Candida n* (%)**		50 (6.4)	46 (9.4)	27 (2.6)	277 (8.2)	12 (2.5)	92 (5.3)	1001 (6.5)
* C. dubliniensis*		4 (0.5)	18 (3.7)	4 (0.4)	144 (4.3)	2 (0.4)	46 (2.7)	264 (1.7)
* C. guilliermondii*		13 (1.7)		7 (0.7)	14 (0.4)		8 (0.5)	91 (0.6)
* C. kefyr*		4 (0.5)	5 (1.0)	3 (0.3)	21 (0.6)	3 (0.6)	7 (0.4)	94 (0.6)
* C. lipolytica*		4 (0.5)	1 (0.2)			1 (0.2)	1 (0.1)	10 (0.1)
* C. lusitaniae*		10 (1.3)	10 (2.1)	8 (0.8)	41 (1.2)	2 (0.4)	25 (1.5)	277 (1.8)
* C. metapsilosis*		2 (0.3)			4 (0.1)			33 (0.2)
* C. orthopsilosis*		7 (0.9)	2 (0.4)		8 (0.2)			82 (0.5)
* C. pelliculosa*		2 (0.3)	4 (0.8)		9 (0.3)		2 (0.1)	22 (0.1)
Other *Candida* spp.		4 (0.5)	6 (1.2)	5 (0.5)	36 (1.1)	4 (0.8)	3 (0.2)	128 (0.8)
**Other yeasts, *n* (%)**	167 (7.7)	15 (1.9)	2 (0.4)	14 (1.4)	46 (1.4)	28 (5.9)		
* Cryptococcus* spp.	109 (5.1)	5 (0.6)	1 (0.2)	5 (0.5)	14 (0.4)	3 (0.6)		
* Trichosporon* spp.	23 (1.1)	3 (0.4)			1 (0.03)	4 (0.8)		
* Rhodutorula* spp.	10 (0.5)	2 (0.3)		2 (0.2)	3 (0.1)	12 (2.5)		
* M. capitatus * ^4^		3 (0.4)			4 (0.1)			
* M. clavatus * ^5^				3 (0.3)	2 (0.1)			
* K. (Pichia) ohmeri * ^6^	7 (0.3)	1 (0.1)						
* Malassezia* spp.	4 (0.2)							
* L. elongisporus * ^7^		1 (0.1)			1 (0.03)			
* E. dermatitidis * ^8^				1 (0.1)				
* S. cerevisiae * ^9^			1 (0.2)	3 (0.3)	18 (0.5)	9 (1.9)		
**Other spp. (no ID)**	14 (0.6)				3 (0.1)			

^1^ In supplement to classical morphology. ^2^ Yeast isolates with known and previously used *Candida* anamorphs have for comparison all been named with their *Candida* names, even if they now would be considered in other genera (e.g., *Pichia*, *Wickerhamomyces*, *Metschnikowia*, *Yarrowia,* etc.). Grey shading is used if information is not available. Colour codes are used to highlight differences in proportional species distribution as follows: <0.5% (light yellow); 0.5–0.9% (light orange), 1–5% (light green), 6–10% (green); >10% (blue). Fields left blank when no isolates of a given species were found. ^3^ For studies carried out in Greece (stated) and presumably Lombardy and Norway, only *C. parapsilosis* and *C. glabrata* species complex are named. ^4^ *Magnusiomyces capitatus.* ^5^ *Magnusiomyces capitatus/Saprochaeta clavata*. ^6^ *Kodamaea ohmeri*. ^7^ *Lodderomyces elongisporus*. ^8^ *Exophiala dermatitidis*. ^9^ *Saccharomyces cerevisiae*.

## 3. Variation of MICs and Epidemiological Cut-Off Values

In vitro resistance may be primary (innate, inherent, intrinsic) or secondary (acquired). Primary resistance is the natural resistance of a fungal order, genus, or species to an antifungal class or a single antifungal agent as a consequence of functional and structural characteristics. In contrast, acquired resistance describes the situation when isolates from a normally susceptible species become less susceptible or resistant, owing to molecular mechanisms such as target gene mutations or target gene or efflux pump upregulation [28,29].

**Figure 1:**
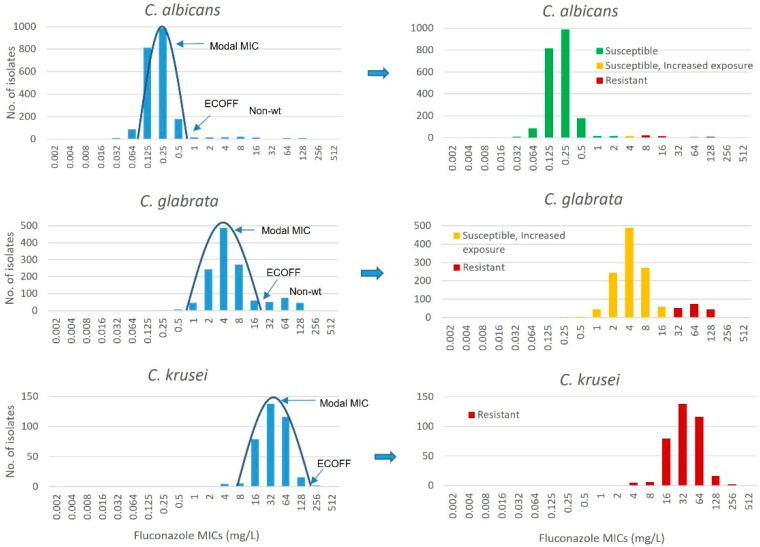
MIC distributions with applied ECOFFs, clinical breakpoints, and susceptibility classifications for three common *Candida* species. MIC distributions, ECOFFs, and clinical breakpoints are based on the EUCAST document “Fluconazole: Rationale for the clinical breakpoints,” version 3.0, 2020. http://www.eucast.org (accessed on 9 December 2021).

Because of inherent variation associated with phenotypic susceptibility testing, MICs of wild-type isolates (without acquired resistance) of a given species group together in a bell-shaped Gaussian distribution within a range of +/− one to two two-fold dilution steps around a “modal” (most common) MIC (Figure 1). The modal MIC represents the “true” MIC of the species [30]. With a normal Gaussian distribution, the MIC_50_ (the MIC that encompasses 50% of isolates) will be identical to the modal MIC. The epidemiological cut-off value (abbreviated as ECOFF by EUCAST and ECV by CLSI) is the MIC/MEC that defines the upper limit of the MICs of the phenotypical wild-type population. MICs higher than those encompassed within the Gaussian distribution cannot be explained by inherent variation but represent isolates with acquired resistance mechanisms. The clinical implication of such elevated MICs depends on the dose that can be given. Isolates with acquired resistance may form a hump, a tail, or a second Gaussian distribution to the right, depending on the number of resistant isolates and the magnitude of the MIC elevation (Figure 1).

## 4. The Difference between ECOFFs and Clinical Breakpoints

ECOFFs are defined as the highest MIC value for wild-type isolates (isolates without phenotypically detectable acquired and mutational resistance mechanisms to the agent in question) and are determined solely on the basis of MIC distributions. EUCAST set criteria for setting ECOFFs, including minimum number of data sets, minimum number of isolates per data set and overall, and rules for qualifying data sets as acceptable or not and defined several ECOFFs or tentative ECOFFs (tECOFFs) (Table 2) [31]. ECOFFs alone neither provide a susceptibility interpretation nor work as an indicator for therapeutic outcome as wild-type isolates can be susceptible, susceptible Increased exposure, or resistant, depending on the species, drug, and dose that can be achieved in the patient (Figure 1). However, ECOFFs allow classification of isolates as wild-type, i.e., “normal” (meaning the clinician can rely on his/her experience with that particular microorganism and assume that existence of secondary resistance-related mutations is not likely) or non-wild-type (meaning the isolate has acquired resistance mechanisms and may not respond as well as other isolates in that species).

Clinical breakpoints incorporate knowledge about wild-type MIC distributions, dosing, clinical outcome data (clinical trials and in vitro animal experiments), and pharmacodynamics/pharmacokinetic parameters [32,33]. When available, knowledge about target gene mutations and their clinical implications is also taken into account. Ideally, it should be clearly demonstrated that a licensed dosage will give an exposure sufficient to achieve the optimal PK/PD index for wild-type isolates (with MICs up to the ECOFF) and result in a good clinical outcome. The breakpoints are established to identify isolates with acquired resistance conferring less likelihood for treatment efficacy. Importantly, as inherent variation in susceptibility testing explains the MIC variation within the wild-type population (end thus below the ECOFF), breakpoints should be set without bisecting the wild-type distribution as this would lead to random classification of wild-type isolates. In most cases the breakpoint is the same value as the ECOFF for susceptible species, either because the standard dose has been selected as the lowest dose that safely covers wild-type isolates (to limit the risk of unnecessary toxicity) or because sufficient evidence is not available to determine what degree of MIC elevation can occur without negatively affecting efficacy.

**Table 2:** Overview of the species and compounds for which EUCAST ECOFFs or tentative ECOFFs (tECOFFs indicated in brackets) have been established for yeast species [1]. A dash denotes that an ECOFF has not been established for that particular organism and drug.

Species	MIC mg/L									
	AMB	CAS ^1^	MFG	AFG	FLC	VRC	ITC	POS	ISA	5FC
*C. albicans*	1	-	0.016	0.03	0.5	0.03	0.06	0.06	-	-
*C. dubliniensis*	0.25	-	-	-	[0.5]	0.03	0.06	0.06	-	-
*C. glabrata*	1	-	0.03	0.06	16	1	2	1	-	-
*C. guilliermondii*	[0.5]	-	-	-	[16]	-	2	0.25	-	-
*C. kefyr*	[1]	-	-	-	[1]	-	-	-	-	-
*C. krusei*	1	-	0.25	0.06	128	1	1	0.5	-	-
*C. lusitaniae*	[0.5]	-	-	-	-	-	0.125	-	-	-
*C. parapsilosis*	1	-	2	4	2	0.06	0.125	0.06	-	-
*C. tropicalis*	1	-	0.06	0.06	1	0.125	0.125	0.06	-	-
*Cryptococcus neoformans*	[1]	-	-	-	-	0.5	-	0.5	-	-
*Cryptococcus gattii*	[0.5]	-	-	-	-	-	-	1	-	-
*S. cerevisiae*	[0.5]	-	-	-	-	-	-	-	-	-

^1^ EUCAST does not currently recommend susceptibility testing of caspofungin because of significant inter-laboratory variation in MIC ranges. AMB: Amphotericin B, CAS: Caspofungin, MFG: Micafungin, AFG: Anidulafungin, FLC: Fluconazole, VRC: Voriconazole, ITC: Itraconazole, POS: Posaconazole, ISA: Isavuconazole. 5FC: Flucytosine.

## 5. Pragmatic Guidance for MIC Interpretation in the Absence of Breakpoints

### 5.1. General Considerations

For many rare yeasts, sufficient data neither for ECOFF nor for breakpoint setting have been available. Until breakpoints become available our pragmatic conceptual approach and resulting recommendations in Table 3 regarding “how to interpret the MIC” are described in the following section.

First, we regard it important to ascertain that the species identification is correct (MALDI-TOF or sequencing) and to re-test the isolate once or twice to confirm the MIC is representative for the isolate, as the inherent variability in MIC determinations may yield varying MICs within a couple of dilution steps.

Second, we recommend comparing the MICs to existing MIC distributions for that drug and species to determine if the MIC is most likely “normal” or “elevated” (indicating possible acquired resistance). In Table 4, Table 5, Table 6 and Table 7, we summarised our data for more than 4000 isolates and documented alignment to published EUCAST data [25,34,35,36,37,38,39,40,41,42,43,44,45,46,47,48,49,50,51,52] (for details on this data, see Supplementary Text S2). These tables can be used for comparison if the local susceptibility testing is confirmed to align with EUCAST testing.

Third, we propose a pragmatic categorisation and upper limits of the wild-type MICs for each of the rare species. Our categorisation relies on a comparison to the modal MIC and range obtained for (preferably related) common species. The underlying assumptions are, firstly, that when isolates are genetically related, pathogenicity, invasive potential, propensity for plastic adherence, and intrinsic resistance mechanisms may be expected to be similar as well. Secondly, even if species are not related phylogenetically, we pragmatically assumed that the rare yeasts are most likely “rare” due to a lower rather than higher pathogenicity and therefore will likely respond if the MIC is comparable to that against wild-type isolates of more prevalent and susceptible species. The categorisation is presented in Table 4, Table 5, Table 6 and Table 7 and summarised with pragmatic “breakpoints” (BPs) in Table 3.

### 5.2. Amphotericin B

For amphotericin B, all included genera except *Trichosporon* generally had MICs below the non-species-specific clinical susceptibility breakpoint of 1 mg/L for *Candida* species in agreement with the broad clinical spectrum of this agent (Table 4). All *Cryptococcus neoformans* isolates had MICs below 1 mg/L and the recommended first line treatment for this organism is indeed amphotericin B (+/− flucytosine) [53]. One isolate of *C. lipolytica* (*Yarrowia lipolytica*) had an MIC of 2 mg/L but grew very poorly, likely influencing the reading. Another series of 27 *C. lipolytica* isolates reported an MIC_90_ of 1 mg/L [45]. It is therefore reasonable to assume that MICs are normally ≤1 mg/L. *Candida lusitaniae* (*Clavispora lusitaniae*) does not normally have MIC values above 1 mg/L, but as a lower fungicidal activity, higher mutation rates, and clinical failure rates were seen, amphotericin B is not recommended for treatment for this species [34,54]. For *Trichosporon* species, higher MICs are common, and amphotericin B is not the recommended first line treatment (Table 4). Based on this, we would pragmatically regard yeast isolates with amphotericin B MIC ≤ 1 mg/L as wild-type and regard them as a suitable target for amphotericin B, with the exception of *C. lusitaniae* and *Trichosporon* species isolates (Table 3).

**Table 3:** Overview of pragmatic BPs for the rare yeast species. The colour denotes the intrinsic relative susceptibility (S (green)/I (orange)/R (red)/Unknown (grey)) of wild-type isolates (isolates with modal MICs (mg/L) in the indicated range). Isolates with MICs above the indicated species-specific values (and thus non-wild-type) should be regarded resistant.

Recommendation Regarding Treatment	Amphotericin B	Anidulafungin	Fluconazole	Voriconazole
Treat if wild-type	Confirmed MIC ≤ 1: *Candida* species Rare yeasts (except those below)	Confirmed MIC ≤ 0.06: →regard susceptible *C. dubliniensis* *C. inconspicua* *C. nivariensis* *C. norvegensis* *C. pelliculosa* *C. utilis* *L. elongisporus* *P. kluyveri* Repeat MIC ≤0.125 mg/L →regard susceptible (consider *FKS* sequencing if MIC > 0.06 mg/L) *C. intermedia* *C. lusitaniae* *C. palmioleophila* *C. kefyr*	Confirmed MIC ≤ 2: →regard susceptible *C. intermedia* *C. kefyr* [1] *C. lusitaniae* *C. metapsilosis* *C. orthopsilosis* *C. utilis* *L. elongisporus*	Confirmed MIC ≤0.03: →regard susceptible *C. intermedia* *C. kefyr* *C. lusitaniae* *C. metapsilosis* *C. orthopsilosis* *L. elongisporus*
Consider use if wild-type and: Not severe/ Elevated dose/ Oral consolidation/ No better options		Confirmed MIC 0.125–0.5: →consider use in some situations (for ex. less severe infections, when no better option is available) *C. lipolytica* *C. magnoliae* *C. metapsilosis* *C. orthopsilosis* *C. pararugosa* *S. cerevisiae* *A. adeninivorans*	Confirmed MIC 2–16: →consider use in some situations (increased dosage and less severe infections) *C. fermentati* *C. nivariensis* *C. pararugosa* *C. pelliculosa* *C. guilliermondii* [16] *C. bovina* *T. dermatis* (1st line Alt) *Cr. Neoformans* (2nd line) *S. cerevisiae* *T. asahii* (1st line Alt)	Confirmed MIC 0.06–0.125: →consider use in some situations (TDM confirmed sufficient exposure, less severe infections or when no better option is available) *C. fermentati* *C. guilliermondii* *C. lipolytica* *C. nivariensis* *C. palmioleophila* *C. pelliculosa* *C. utilis* *S. cerevisiae* *Cr. neoformans* [0.5] *T. dermatis* (1st line)
Consider alternative therapy	Confirmed MIC >1: Any isolate →regard resistant *C. lusitaniae* [0.5] *Trichosporon* spp. (2nd line)	Repeat MIC 0.5–1 No evidence that allows recommendation *C. fermentati* *C. guilliermondii* Repeat MIC ≥1: →regard resistant *Cryptococcus* *Trichosporon*, *Magnusiomyces*, *Geotrichum and Rhodutorula* (Against due to intrinsic resistance)	Confirmed MIC > 16 →regard resistant *C. inconspicua* *C. lipolytica* *C. magnoliae* *C. norvegensis* *C. palmioleophila* *P. kluyveri* *G. candidum* *R. mucilaginosa* *M. capitatus* *M. clavatus* *A. adeninivorans*	Confirmed MIC 0.25–1: No evidence that allows recommendations *C. inconspicua* *C. norvegensis* *P. kluyveri* *M. capitatus* (1st line Alt) *G. candidum* (1st line Alt) Confirmed MIC testing ≥2 →Regard as resistant *A. adeninivorans* *R. mucilaginosa* (Against)

Only species with >1 isolate are included. tECOFFs in brackets. Treatment recommendations from the 2021 Global ECMM/ISHAM/ASM Rare Yeast Guideline [14] (or 2010 IDSA guideline for meningitis/cryptococcemia [53]) are used in parenthesis where available. “1st line Alt” refers to first line alternative.

**Table 4:** Amphotericin B EUCAST MICs against Danish common and rare yeast species sorted by increasing MICs. The coloured boxes indicate the intrinsic relative susceptibility (S (green)/I (orange)/R (red)) of wild-type isolates of a given species.

Species	n	Amphotericin MIC (mg/L)									ECOFF/ WT susc. ^1^	ECMM/ISHAM/ASM Recommendation (SoR/QoE) [14] ^2^	Number; MIC_50_/MIC_90_ (Range), [Reference] ^3^
		≤0.016	0.03	0.06	0.125	0.25	0.5	1	2	4			
*C. dubliniensis*	235	26	57	**102**	41	9					0.25/S		n = 146; 0.06/0.25 [34]
*C. albicans*	1260		2	81	337	**707**	132	1			1/S		n = 1342; 0.25 /0.5 [34]
*C. glabrata*	947		6	28	149	**433**	323	8			1/S		n = 907; 0.25/0.5 [34]
*C. tropicalis*	147				5	**75**	64	3			1/S		n = 257; 0.25/0.5 [34]
*C. krusei*	150				1	3	**73**	**73**			1/S		n = 262; 0.5/1 [34]
*C. parapsilosis*	128				3	35	**81**	9			1/S		n = 314; 0.5/0.5 [34]
*P. kluyveri*	2		2										
*C. intermedia*	1			1									n = 13; 0.25/1 [45], n = 1, (0.03) [46]
*P. manshurica*	1				1								n = 1; (0.25) [47]
*L. elongisporus*	2				2								n = 1, 0.03 [49]; n = 2 (0.03-0.12) [46]
*C. pararugosa*	2				1		1						n = 60; 1/1 [48], n = 6; 1/1 [45]
*C. utilis*	3				1	2							
*C. fermentati*	11			1	4	**6**							n = 29; 0.5/2 [45]
*C. pelliculosa*	12			3	3	**4**	2						n = 30; 0.5/1 [45]
*R. mucilaginosa*	7			1	1	**3**	2					1st line (+/− 5FC) (BIIu/BIII)	n = 1; (0.25) [49]; n = 5; (0.5–1) [50]
*C. guilliermondii*	32			2	14	**15**		1			[0.5]		n = 88; 0.125/0.25 [34], n = 30, 0.125/0.25 [51], n = 27; 1/1 [45]
*C. lusitaniae*	61			6	24	**26**	4	1			[0.5]		n = 59; 0.125/0.25 [34], n = 30; 0.06/0.25 [51], n = 14; 0.25/1 [45]
*C. orthopsilosis*	15				2	6	7						n = 5; 0.06/NA (0.03-0.12) [25]; n = 8; (0.03-0.12) [46]
*Cr. neoformans* SC	17		1	1	2	**9**	3	1			[1]	1st line (+/− 5FC) (IDSA)	n = 1022; 0.25/0.5 [34], n = 106, 0.125/0.25 [52]
*S. cerevisiae*	58		1	4	11	**26**	15	1			[0.5]	1st line (BIII)	n = 81; 0.25/0.5 [34]
*C. palmioleophila*	1					1							n = 3; (0.125-0.5) [45]
*C. fabianii*	1					1							n = 2, (0.06-0.25) [46]
*C. inconspicua*	6				1		3	2					n = 168; 0.5/1 [48]; n = 5 (0.25-0.5) [46]
*C. kefyr*	47					9	**32**	6			[1]		n = 64; 0.5/1 [34], n = 17; 1/2 [45]
*C. nivariensis*	4					1	3						n = 4; 0.125/0.25 [45]
*K. ohmeri*	1						1					1st line (BIII)	n = 1, 0.03 [49], n = 1; (0.125) [50]; n = 4 (0.03-0.12) [46]
*C. catenulata*	1						1						n = 1; (0.06) [47]
*C. norvegensis*	10					1	**7**	2					n = 18; 0.25/1 [45], n = 15; 1/2 [48]
*C. metapsilosis*	5					**4**	1						n = 6; 0.09/NA (0.06-0.12) [25]
*C. lipolytica*	2						1		1				n = 27; 0.5/1 [45]
*C. ciferrii*	1							1					n = 8; (1-2) [48]; n = 1, 0.25 [46]
*A. adeninivorans*	3						2	1					n = 1; (2) [50]
*G. candidum*	4						2	2				1st line (+/− 5FC) (BIII)	n = 3; (0.25-1) [35]
*M. capitatus*	11						1	**9**	1			1st line (+/− 5FC) (BIIu)	n = 27; 0.25/0.5 [35], n = 3 (0.125-0.5) [49]
*T. asahii*	1							1				2nd line (CIIu)	n = 37; 2/16 [36], n = 29 (0.25-4) [37], n = 2 (2) [49], n = 1; (>8) [50]
*T. dermatis*	2							1		1		2nd line (CIIu)	n = 1; (2) [36], n = 1 (0.13), [37], n = 1; (1) [50]
*T. inkin*	1									1		2nd line (CIIu)	n = 3; (0.25-1) [37], n = 2; (>8) [50]

^1^ ECOFFs (tentative ECOFFs in brackets), wild-type susceptibility classifications (common species). ^2^ Treatment recommendations (Strength of Recommendation (SoR) and Quality of Evidence (QoE)) from the 2021 Global ECMM/ISHAM/ASM Rare Yeast Guideline are included for comparison for all except *Cr. neoformans*. For *Cr. neoformans*, the 2010 IDSA guideline for meningitis/cryptococcemia is used [53]. ^3^ Available published EUCAST MIC distributions from EUCAST or other laboratories were retrieved and referenced to the right for comparison (range used when few isolates were reported). EUCAST clinical breakpoints for the common species inserted in solid line/dotted line. The MIC_50_ is underlined (species with >10 isolates) and the modal MIC is in bold (≥5 isolates). For isolates with ≥10 isolates, the distribution around the modal MIC/MIC_50_ is marked in shaded grey. S/R classification in green/red. NA: Not available. The data set includes the MIC values for candidaemia and non-bloodstream isolates as specified in the Supplementary Text S2.

### 5.3. Anidulafungin

For anidulafungin, as a marker for echinocandin resistance, a broader range of MIC distributions were found. Most rare *Candida* and *Pichia* species had modal MICs ≤0.06 mg/L, whereas isolates of *Cryptococcus*, *Magnusiomyces*, *Geotrichum*, *Trichosporon* and *Rhodutorula* all had high MICs (≥1 mg/L) and are considered intrinsically resistant in accordance with recommendations against echinocandin therapy [4,5,53]. Resistance to the echinocandins in *Candida* spp. is almost exclusively associated with amino acid alterations in two hotspots in the target genes *FKS1* (and for *C. glabrata* also *FKS2*). Acquired resistance has been detected in various species normally considered echinocandin susceptible, such as *C. albicans*, *C. dubliniensis*, *C. glabrata*, *C. krusei*, *C. tropicalis*, *C. kefyr* (*Kluyveromyces marxianus*) and *C. lusitaniae* (*Clavispora lusitaniae*) [28]. *C. auris* isolates can also harbour *FKS* mutations [55]. For the rare species, we have *FKS* data for a few isolates with high MIC for *C. dubliniensis* and *C. kefyr* (Table 5). Fourteen species had modal MICs of 0.016–0.03 mg/L, similar to *C. tropicalis*, *C. glabrata* and *C. krusei*, which all have a clinical breakpoint of 0.06 mg/L (Table 5). MIC ≤0.06 mg/L separated wild-type from non-wild-type isolates of *C. dubliniensis* and included the wild-type populations of *C. inconspicua*, *C. norvegensis* (*Pichia norvegensis*), *C. pelliculosa (Wickerhamomyces anomala*), *C. nivariensis*, *P. kluyveri* and *L. elongisporus*. The modal MICs against *C. intermedia*, *C. palmioleophila*, *C. kefyr* and *C. lusitaniae*, which have been shown to respond to candin therapy unless having acquired an *FKS* mutation [56,57,58], were approximately one two-fold dilution higher, suggesting a tentative threshold for suspicion of resistance of >0.125 mg/L for these species [39,45]. When possible, *FKS* sequencing is, however, recommended for isolates for which repeated MICs are greater than or equal to 0.06 mg/L as this will enable detecting mutations around the likely ECOFF (Table 5).

A group of ten yeast species yielded intermediate modal MICs of 0.125–1 mg/L, including species in the *C. parapsilosis* species complex, *C. guilliermondii* (*Meyerozyma guilliermondii*) and *C. fermentati* (*Meyerozyma caribbica*), which have intrinsic alterations associated with higher inherent MIC values [28,59]. Breakthrough and persistent infections during echinocandin treatment have been observed for all three species, indicating that they are somewhat less susceptible [59,60,61,62,63]. In a French study of fungaemia caused by uncommon yeasts, *C. guilliermondii* fungaemia was also found to be associated with pre-exposure to caspofungin [47]. However, likely because of the lower pathogenicity, no increase in mortality or clinical failure was seen in two retrospective studies of patients with *C. parapsilosis* candidaemia initially treated with an echinocandin [64,65]. As a result, the 2016 IDSA Candida guideline states that in specific cases (clinically stable patients and if follow-up culture results are negative), continuing use of the echinocandin until completion of therapy is reasonable [66]. The EUCAST clinical breakpoints for *C. parapsilosis* have also been updated so that wild-type isolates of *C. parapsilosis* are now classified as susceptible [1]. For some rare yeast species, alternative treatment options are hampered by intrinsic resistance to antifungal drugs other than echinocandins, namely, azoles or amphotericin B, and this must also be considered alongside drug-related side effects and interactions with co-medications. We therefore recommend that for isolates of *C. orthopsilosis*, *C. metapsilosis*, *C. pararugosa* (*Wickerhamiella pararugosa*), *C. magnolia*, *C. lipolytica* (*Yarrowia lipolytica*), *S. cerevisiae* and *Arxula adeninivorans* with MIC 0.125–0.5 mg/L, anidulafungin treatment is to be considered for less severe infections or if necessitated by drug–drug interactions, side effects, or resistance to other drug classes (Table 3). For isolates of the closely related species of *C. guilliermondii* (*Meyerozyma guilliermondii)* and *C. fermentati* (*M. caribbica)*, which have even higher modal MICs of 0.5–1 mg/L (similar to *K. ohmeri*), we found that the clinical data do not support any definitive recommendation regarding echinocandin monotherapy. Of note, *C. fermentati* has been associated with several breakthrough infections during echinocandin therapy suggesting alternative or combination therapy should be considered, particularly for invasive infections [59,62].

**Table 5:** Anidulafungin EUCAST MICs against Danish common and rare yeast species sorted by increasing MICs. The coloured boxes indicate the intrinsic relative susceptibility (S (green)/I (orange)/R (red)/Unkown (grey)) of wild-type isolates of a given species.

Species	n	Anidulafungin MIC (mg/L)									EUCAST ECOFF/WT susc. ^1^	ECMM/ISHAM/ASM recommendation (SoR/QoE) [14] ^2^	Number, MIC_50_/MIC_90_ (Range), [Reference] ^3^
		≤0.008	0.016	0.03	0.06	0.125	0.25	0.5	1	>1			
*C. albicans*	1928	**1626**	255	44	2			1			0.03/S		n = 958; 0.004/0.016 [38]
*C. tropicalis*	200	41	**89**	56	13	1					0.06/S		n = 110; 0.016/0.03 [38]
*C. glabrata*	1351	52	352	**591**	327	11	7	3	5	3	0.06/S		n = 392; 0.016/0.03 [38]
*C. krusei*	204	4	27	**102**	63	6		1		1	0.06/S		n = 60; 0.016/0.06 [38]
*C. parapsilosis*	164						5	43	**77**	39	4/S		n = 419; 1/2 [38]
*C. dubliniensis*	276	107	**130**	36	3 ^4^	1 ^5^	2 ^5^						n = 30; (≤0.016) [51], n = 14; 0.03/0.06 [39], n = 7; (0.03) [46]
*P. kluyveri*	2	2											
*C. inconspicua*	10	4	**4**	2									n = 168; 0.03/0.06 [48], n = 5; 0.03 [46]
*C. norvegensis*	10	1	**6**	3									n = 18; 0.016/0.06 [45], n = 15; 0.03/0.125 [48]
*C. pelliculosa*	14	4	**10**										n = 30; 0.008/0.016 [45]
*C. utilis*	4	3			1								
*C. nivariensis*	6		**3**	1	2								n = 4; 0.016/0.03 [45]
*L. elongisporus*	2		2										n = 1; 0.03 [49], n = 2, 0.03 [46]
*C. intermedia*	3		1	2									n = 13; 0.03/0.125 [45], n = 1; 0.03 [46]
*C. ciferrii*	1			1									n = 8; (0.03->4) [48]
*C. fabianii*	1				1								n = 2; (0.03) [46]
*C. palmioleophila*	10		2	**6**	1	1							n = 3; (0.03-0.5) [45]
*C. kefyr*	56	1	13	**31**	8	2^4^				1 ^5^			n = 17; 0.03/0.125 [45]; n = 8; 0.06/0.125 [39]
*C. lusitaniae*	76		3	**31**	29	11	2						n = 24; 0.125/0.5 [39], n = 30; 0.016/0.125 [51], n = 14; 0.06/0.125 [45]
*S. cerevisiae*	63		1	6	**25**	**25**	5	1				1st line Alt (BIII) ^6^	
*C. metapsilosis*	6				1	**3**	1	1					n = 6; 0.18/NA (0.06-1) [25]
*C. magnoliae*	2					1	1						
*C. pararugosa*	2					1				1			n = 60; 0.5/>4 [48], n = 6; 0.25/0.5 [45]
*A. adeninivorans*	3		1			1		1					n = 1; (0.5) [50]
*C. orthopsilosis*	16					1	**10**	2	3				n = 27; 2/2 [40], n = 5; (0.25-0.5) [25]; n = 8; (0.12-1) [46]
*C. lipolytica*	2							2					n = 27; 0.25/0.5 [45]
*C. fermentati*	24						4	**10**	7	3			n = 29; 1/2 [45]
*K. ohmeri*	1								1			1st line Alt (BIIu/BIII) ^6^	n = 1; (1) [49], n = 4; (0.03-4) [46], n = 1; (1) [50]
*C. guilliermondii*	58					2	4	15	**23**	14			n = 32; 1/2 [38], n = 30; 0.5/2 [51], n = 27; 1/2 [45], n = 8; 2/4 [39]
*Cr. neoformans* SC	21									**21**		Against (IDSA)	
*M. capitatus*	11								1	**10**		Against (DIIu-DIII)	n = 3, (2-32) [49]
*M. clavatus*	2									2		Against (DIIu-DIII)	
*G. candidum*	4								1	3		Against	
*T. asahii*	2									2		Against	n = 2, (4-32) [49], n = 1; (>8) [50]
*T. dermatis*	2									2		Against	n = 1; (>8) [50]
*T. inkin*	1									1		Against	n = 2; (>8) [50]
*R. mucilaginosa*	8									8		Against	n = 1; 32 [49], n = 5 (>8) [50]

^1^ ECOFFs (tentative ECOFFs in brackets), wild-type susceptibility classifications (common species). ^2^ Treatment recommendations (Strength of Recommendation (SoR) and Quality of Evidence (QoE)) from the 2021 Global ECMM/ISHAM/ASM Rare Yeast Guidelines are included for comparison for all except *Cr. neoformans*. For *Cr. neoformans*, the 2010 IDSA guideline for meningitis/cryptococcemia is used [53]. “1st line Alt” refers to first line alternative [14]. ^3^ Available published EUCAST MIC distributions from EUCAST or other laboratories were retrieved and referenced to the right for comparison (range used when few isolates were reported). ^4^ These isolates were *FKS* WT. Micafungin MICs were: ≤0.03 mg/L (*C. dubliniensis*)/0.06 mg/L (*C. kefyr*). ^5^ Isolates with demonstrated *FKS* mutations. ^6^ No SoR/QoE data for anidulafungin, only for micafungin and caspofungin; therefore, only these are recommended for *S. cerevisiae* (and *K. ohmeri*). EUCAST clinical breakpoints for the common species inserted in solid line/dotted line. The MIC_50_ is underlined (species with ≥10 isolates) and the modal MIC is in bold (≥5 isolates). For isolates with ≥10 isolates, the distribution around the modal MIC/MIC_50_ is marked in shaded grey. S/I/R classification in green/orange/red. NA: Not available. The data set includes the MIC values for candidaemia and non-bloodstream isolates as specified in the Supplementary Text S2.

### 5.4. Fluconazole

For fluconazole, EUCAST has the same species-specific breakpoint of 2 mg/L for the four common susceptible species, based on clinical outcome data, microbiological data, dosing and PK data. A similar non-species-specific clinical fluconazole breakpoint supported by PK/PD data has been established, and most isolates of species like *C. kefyr*, *C. lusitaniae*, *C. metapsilosis*, *C. orthopsilosis* and *L. elongisporus* would thus be considered susceptible (Table 6). Other species like *C. fermentati* (*Meyerozyma caribbica*), *C. nivariensis* (closely related to *C. glabrata*), and *S. cerevisiae* (closely related to *C. glabrata*), *Cryptococcus neoformans*, *C. guilliermondii,* and *C. pelliculosa* (*Wickerhamomyces anomalus*) had higher modal MICs of 2–8 with MIC ranges straddling the susceptibility breakpoint, similar to *C. glabrata,* which is considered susceptible given increased exposure [1]. In support of this, increased dosing is recommended in clinical practice for *C. glabrata* and when used for consolidation treatment of *C. neoformans* infections [13,53]. Finally, isolates of *C. palmioleophila*, *C. norvegensis*, *C. inconspicua (Pichia cactophila)*, *C. lipolytica*, *Magnusiomyces* spp, *Pichia kluyveri*, *G. candidum*, *R. mucilaginosa*, *A. adeninivorans* and *Trichosporon asahii* had modal MICs of 16-≥32 mg/L, similar to the intrinsically resistant species *C. krusei,* and it would thus appear advisable to seek alternative treatment (Table 3 and Table 6). Of note, our included isolates of three *Trichosporon* species (*T. inkin*, *T. dermatis* (*Cutaneotrichosporon dermatis*) and *T. asahii*) displayed stepwise increasing MICs (Table 6). This was based on few isolates but aligned with the note that the MICs may vary among *Trichosporon* species in the recent Rare Yeast Guideline and that fluconazole is moderately recommended provided the MIC is low. In the clinical studies, fluconazole 400–800 mg has been used, which also indicates that elevated dosing may be recommendable [14].

**Table 6:** Fluconazole EUCAST MICs against Danish common and rare yeast species sorted by increasing MICs. The coloured boxes indicate the intrinsic relative susceptibility (S (green)/I (orange)/R (red)) of wild-type isolates of a given species.

Species	n	Fluconazole MICs (mg/L)									EUCAST ECOFF/ WT susc. ^1^	ECMM/ISHAM/ASM Recommendation (SoR/QoE) [14] ^2^	Number, MIC_50_/MIC_90_ (Range) ^3^
		≤0.125	0.25	0.5	1	2	4	8	16	≥32			
*C. albicans*	1972	**927**	895	120	14	4	4	1		7	0.5/S		n = 2175; 0.25/0.5 [41]
*C. dubliniensis*	280	**116**	85	49	15	2	1	1	3	8	[0.5]/S		n = 142; 0.25/0.5 [41]
*C. tropicalis*	203	15	57	**71**	44	2	5	2	3	4	1/S		n = 551; 0.5/2 [41]
*C. parapsilosis*	171		9	**87**	54	12	3	1	1	4	2/S		n = 835; 0.5/2 [41]
*C. glabrata*	1385		1	3	38	377	**671**	117	27	**151**	16/I		n = 1289; 4/32 [41]
*C. krusei*	206							5	39	**162**	128/R		n = 363; 32/64 [41]
*L. elongisporus*	2		2										n = 7, (≤0.125-0.5) [42]; n = 1, 0.25 [49]; n = 2; (0.12) [46]
*C. kefyr*	57	3	20	**23**	8	2		1			[1]		n = 170; 0.25/1 [42], n = 69; 0.25/1 [41], n = 17; 0.5/2 [45], n = 8; 0.5/16 [39]
*C. lusitaniae*	77	5	24	**36**	5	1			2	4			n = 221, 0.25/0.5 [42], n = 30; 0.25/2 [51], n = 24; 0.25/1 [39], n = 14, 0.25/1 [45]
*C. intermedia*	3			1	1	1							n = 13; 0.5/1 [45], n = 1; (0.25) [46]
*C. fabianii*	1			1									n = 10; 0.5/1 [42], n = 2; (0.5-1) [46]
*T. inkin*	1				1							1st line Alt (BIIu)	n = 10; 2/4 [42], n = 3; (2) [37], n = 2; (1) [50]
*C. metapsilosis*	6				3	3							n = 45; 1/2 [42], n = 9; (0.5-8) [46], n = 6; 1/NA [25]
*C. orthopsilosis*	16		2	**4**	3	2			1	4			n = 49; 0.5/8 [42], n = 5; (0.5) [25], n = 8; (0.5) [46]
*C. utilis*	4				1	2			1				n = 23; 1/4 [42]
*C. catenulata*	1					1							n = 1; (0.5) [47]
*T. dermatis*	2					1	1					1st line Alt (BIIu)	n = 7; (1-≥64) [42], n = 1; (0.25) [36], n = 1; (2) [37], n = 1; (4) [50]
*C. fermentati*	24				4	**11**	4		2	3			n = 35; 8/≥64 [42]¸n = 29; 16/32 [45]
*C. nivariensis*	6					2	**4**						n = 13; 4/4 [42], n = 4; 4/16 [45]
*C. pararugosa*	2						1		1				n = 60; 16/64 [48], n = 9; (4-16) [42] n = 6, 16/>64 [45]
*C. pelliculosa*	14					6	**7**	1					n = 36; 2/4 [42], n = 30; 4/8 [45]
*Cr. neoformans* SC	21				1	3	**7**	6	4			2nd line (IDSA)	n = 106; 4/16 [52], n = 1126; 4/8 [42] ^4^
*C. guilliermondii*	59					6	**18**	**21**	2	12	[16]		n = 115; 8/≥64 [42], n = 66; 4/128 [41], n = 30; 2/16 [51], n = 27; 8/16 [45], n = 8; 4/128 [39]
*S. cerevisiae*	64					4	20	**24**	10	6		1st line Alt (BIIu)	n = 61; 8/16 [42]
*K. ohmeri*	1							1				1st line Alt (BIIu-BIII)	n = 32; 4/16 [42], n = 4; (2-8) [46], n = 1; (8) [49], n = 1; (16) [50]
*C. bovina*	1								1				n = 5; (2-8) [42]
*T. asahii*	2								2			1st line Alt (BIIu)	n = 59; 4/16 [42], n = 37; 8/64 [36], n = 29; (1-64) [37], n = 2; (1-4) [49], n = 1; (16) [50]
*M. capitatus*	11						2	**3**	**3**	**3**		(BIIu-DIII)	n = 56; 8/16 [42], n = 28; 4/16 [35] , n = 3, (32-128) [49]
*C. palmioleophila*	10						1	1	3	**5**			n = 20; 8/32 [42], n = 3; (8-16) [45]
*R. mucilaginosa*	8									**8**		Against (BIIu-DIII)	n = 55; ≥64/≥64 [42], n = 5; (64) [50], n = 1; (128) [49]
*C. norvegensis*	11								5	**6**			n = 19; 32/≥64 [42], n = 18; 32/64 [45] , n = 15; 64/>64 [48]
*C. inconspicua*	10								3	**7**			n = 168; 32/>64 [48], n = 45; 16/32 [42], n = 5, (32->64) [46]
*C. lipolytica*	2						1			1			n = 27; 16/32 [45], n = 27; 4/16 [42]
*M. clavatus*	2								1	1		(BIIu-DIII)	n = 184; 16/≥64 [42], n = 18; 8/16 [35]
*C. magnoliae*	2									2			
*C. ciferrii*	1									1			n = 8; (16->64) [48], n = 1; (>64) [46]
*A. adeninivorans*	3									3			n = 1; (64) [50]
*G. candidum*	4									4			n = 36; 16/≥64 [42], n = 3; (2-16) [35]
*P. manshurica*	1									1			n = 1; (64) [47]
*P. kluyveri*	2									2			

^1^ ECOFFs (tentative ECOFFs in brackets), wild-type susceptibility classifications (common species). ^2^ Treatment recommendations (Strength of Recommendation (SoR) and Quality of Evidence (QoE)) from the 2021 Global ECMM/ISHAM/ASM Rare Yeast Guideline are included for comparison for all except *Cr. neoformans*. For *Cr. neoformans*, the 2010 IDSA guideline for meningitis/cryptococcemia is used [53]. “1st line Alt” refers to first line alternative [14]. ^3^ Available published EUCAST MIC distributions from EUCAST or other laboratories were retrieved and referenced to the right for comparison (range used when few isolates were reported). ^4^ MIC_50_/MIC_90_ were 4/8 mg/L for serogroup A and AD, which comprised 949 + 177 = 1126 (84%) of the 1334 isolates, while MIC_50_/MIC_90_ were 1/4 mg/L for the remaining 16% belonging to serogroup D in that study [42]. EUCAST clinical breakpoints for the common species inserted in solid line/dotted line. The MIC_50_ is underlined (species with ≥10 isolates) and the modal MIC is in bold (≥5 isolates). For isolates with ≥10 isolates, the distribution around the modal MIC/MIC_50_ is marked in shaded grey. S/I/R classification in green/orange/red. NA: Not available. The data set includes the MIC values for candidaemia and non-bloodstream isolates as specified in the Supplementary Text S2.

### 5.5. Voriconazole

For voriconazole, common susceptible species have ECOFFs of 0.03 (*C. albicans* and *C. dubliniensis*), 0.125 (*C. tropicalis*) mg/L and breakpoints of 0.06–0.125 mg/L, based on the >72% clinical response to treatment for these species as opposed to 55% for *C. glabrata* [43]. In vitro PK/PD data confirmed the breakpoint at 0.03 mg/L for *C. albicans* based on probability of target attainment and suggested that isolates with MICs of 0.06–0.125 mg/L can only be covered provided sufficient exposure is ensured through therapeutic drug monitoring. However, isolates with MIC ≥ 0.25 mg/L would require trough levels of at least 4 mg/L and thus close to toxic levels [67]. Infections by wild-type isolates of *C. parapsilosis* and *C. tropicalis* had similar outcome data as *C. albicans*, despite slightly higher ECOFFs (0.06 and 0.125 mg/L, respectively) and the limited data for *C. krusei* suggest voriconazole efficacy despite an ECOFF of 1 mg/L [43]. These findings suggested that the lower pathogenicity of most of non-*albicans* species allows efficacy despite some MIC elevation compared to that against *C. albicans*. Based on this, we pragmatically propose considering species with modal MICs of ≤0.03 mg/L as susceptible (repeat MIC of 0.03 mg/L), isolates from species with modal MICs of 0.06–125 mg/L as potentially susceptible (given that an adequate exposure is ensured), isolates with modal MICs 0.25–1 mg/L undefined, and isolates from species with modal MICs >1 as likely resistant (Table 3 and Table 7).

## 6. Interpretation of MICs Obtained by Commercial Tests

Various commercial methods of MIC determination exist, including agar-based MIC gradient strip tests such as the E-test (bioMérieux), automated systems like VITEK 2 (bioMérieux), colorimetric microbroth panels such as Sensititre Yeast One (SYO, Thermo Fisher Scientific), and Micronaut-AM microbroth panels (Merlin–Bruker). MICs determined by these methods may or may not be identical to those generated by reference methods, which should be taken into account before applying breakpoints validated against the reference method [68]. Irrespective of the method used, laboratories should perform an in-house validation of the commercial method and confirm that the commercial method MICs compare to the MICs of the reference method from which the breakpoints are to be adopted. Below we propose a two-step approach for this purpose if EUCAST breakpoints and the recommendations in this review are adopted for *Candida* and rare yeast:

(a) First, test the EUCAST QC strains, 10 times each, and check if the most common MIC (the mode) is on the target and the MICs are within the range (https://www.eucast.org/astoffungi/qcafsttables/). Random variation is permissible (maximum is one MIC value of 10 outside the defined range) but systematic deviation (mode systematically to one side of the target) is not. A systematic deviation shows that breakpoints will not be applicable and needs to be further investigated (concentrations, plastic material, reading of endpoints, etc.). For random variation where more than one MIC is outside the range, continue and perform another 10 tests and allow one of these 10 tests to be out of range.

(b) Second, if the QC strains results agree with the EUCAST QC target and ranges, perform a small study with 10 clinical isolates of the following four common *Candida* species (*C. albicans*, *C. glabrata*, *C. tropicalis,* and *C. krusei*), which will cover different MICs across echinocandins and azoles. It should be confirmed that the mode of each distribution is within ±1 dilution of the mode for each of the drug–bug combinations if compared with the current EUCAST rationale documents (https://www.eucast.org/astoffungi/rationale_documents_for_antifungals/). If they are, EUCAST ECOFFs, breakpoints, and the proposed pragmatic BPs in this review can be adopted. If not, the commercial method in use does not align with the EUCAST method, and consequently, misclassifications are likely to occur.

**Table 7:** Voriconazole EUCAST MICs against Danish common and rare yeast species sorted by increasing MICs. The coloured boxes indicate the intrinsic relative susceptibility (S (green)/I (orange)/R (red)/Unkown (grey)) of wild-type isolates of a given species.

Species	n	Voriconazole MIC (mg/L)												EUCAST ECOFF/WT susc. ^1^	ECMM/ISHAM/ASM Recommendation (SoR/QoE) [14] ^2^	Number, MIC_50_/MIC_90_ (Range) ^3^
		≤0.004	0.008	0.016	0.03	0.06	0.125	0.25	0.5	1	2	4	>4			
*C. albicans*	865	**597**	237	15	7	4				1	1		3	0.03/S		n = 13,630; 0.016/0.03 ^4^ [43]
*C. dubliniensis*	184	40	**106**	26	3	1	2	2					4	0.03/S		n = 101; 0.016/0.03 ^4^ [43]
*C. parapsilosis*	94	2	30	**47**	10	3			1		1			0.06/S		n = 2571; 0.016/0.06 ^4^ [43]
*C. tropicalis*	95		8	34	**40**	6	3			2			2	0.125/S		n = 2958; 0.03/0.125 ^4^ [43]
*C. glabrata*	637			1	71	**340**	131	25	7	17	27	15	3	1/IE		n = 5907; 0.25/1 [43]
*C. krusei*	109						21	**49**	24	11	3		1	1/IE		n = 427; 0.25/1 [43]
*C. kefyr*	43	2	**25**	13	2	1										n = 170; ≤0.015/≤0.015 [42], n = 34; 0.016/0.03 [43]; n = 17; 0.016/0.06 [45], n = 8; 0.016/1 [39]
*C. lusitaniae*	49	5	**33**	8				2	1							n = 221; ≤0.015/≤0.015 [42], n = 91; 0.016/0.06 [43], n = 30; 0.016/0.06 [51], n = 24; 0.016/0.03 [39]
*L. elongisporus*	2	1	1													n = 7; (≤0.015) [42], n = 1; (0.02) [49]
*C. intermedia*	3		1		2 ^6^											n = 13; 0.016/0.03 [45]
*T. inkin*	1				1										1st line (BIIu-CIII)	n = 10; ≤0.015/0.06 [42], n = 3; (0.03-0.13) [37], n = 2; (0.015-0.25) [50]
*C. metapsilosis*	5			2	**3**											n = 45; 0.03/0.06 [42], n = 6; 0.03/NA (0.02-0.12) [25]
*C. catenulata*	1			1												n = 1; (≤0.015) [47]
*C. orthopsilosis*	12		2	**3**	1	2		1		2	1					n = 49; 0.03/1 [42], n = 5; 0.03/NA (0.02-0.03) [25]
*C. nivariensis*	4				2	2										n = 13; 0.06-0.125 [42], n = 4, (0.016-0.125) [45]
*K. ohmeri*	1					1									2nd line (BIII)	n = 32; 0.03/0.125 [42], n = 1, (0.06) [49], n = 1; (0.06) [50]
*C. lipolytica*	2					1						1				n = 27; 0.06/0.125 [42], n = 26;0.125/0.25 [45]
*T. dermatis*	2					2									1st line (BIIu-CIII)	n = 7; (≤0.015-0.125) [42]; n = 1; (0.06) [37], n = 1; (0.03) [36], n = 1 (0.06) [50]
*C. fermentati*	15				3 ^6^	**7**	3		1		1					n = 35; 0.125/2 [42], n = 29; 0.25/0.5 [45]
*C. pelliculosa*	6					1	**4**	1								n = 36; 0.125/0.25 [42], n = 30; 0.06/0.125 [45]
*C. guilliermondii*	50				5 ^6^	**16**	15	2	4	2	2	2	1			n = 125; 0.06/0.5 [43], n = 115; 0.06/0.5 [42], n = 30; 0.06/2 [51], n = 27; 0.125/0.25 [45]
*S. cerevisiae*	48					9	**28**	8	2	1					(BIII)	n = 61; 0.125/0.25 [42], n = 59; 0.125/0.5 [43]
*Cr. neoformans* SC	10			2	1	**3**	**3**	1						0.5		n = 479; 0.125/0.25 [43], n = 106, 0.03/0.06 [52], n = 1126; 0.03/0.125 [42] ^5^
*C. palmioleophila*	9						4	2	1	2						n = 20; 0.125/0.25 [42], n = 3; (0.125) [45]
*C. utilis*	3					1	2									n = 23; 0.06/0.125 [42]
*C. bovina*	1							1								n = 5; (0.03-0.125) [42]
*T. asahii*	1							1							1st line (BIIu-CIII)	n = 59; 0.06/0.25 [42]; n = 37; 1/32 [36], n = 29; (0.03-0.5) [37]; n = 2 (0.25) [49]; n = 1 (0.25) [50]
*C. inconspicua*	4						2	1	1							n = 168; 0.25/1 [48], n = 45; 0.125/0.5 [42]
*G. candidum*	3							1		2					1st line Alt (BIII)	n = 36; 0.25/1 [42], n = 3; (0.06-0.25) [35]
*C. norvegensis*	9						1	**4**	3	1						n = 19; 0.25/0.5 [42], n = 18; 0.5/0.5 [45], n = 15; 1/2 [48]
*M. capitatus*	10					2	2		2	**3**	1				1st line (BIIu)	n = 56; 0.06/0.5 [42], n = 27; 0.125/0.5 [35], n = 3 (1-16) [49]
*C. ciferrii*	1								1							n = 8; (0.5-2) [48]
*C. pararugosa*	1								1							n = 60; 0.5/1 [48], n = 9; (≤0.015-0.25) [42], n = 6, 0.25/0.5 [45]
*P. manshurica*	1								1							n = 1; (0.125) [47]
*P. kluyveri*	2								1	1						
*A. adeninivorans*	2									1			1			n = 1; (1) [50]
*R. mucilaginosa*	5							1		**2**	1	1			Against	n = 55; 2/4 [42], n = 5 (0.5->8) [50], n = 1; (16) [49]

^1^ ECOFFs (tentative ECOFFs in brackets), wild-type susceptibility classifications (common species).^2^ Treatment recommendations (Strength of Recommendation (SoR) and Quality of Evidence (QoE)) from the 2021 Global ECMM/ISHAM/ASM Rare Yeast Guideline are included for comparison. “1st line Alt” refers to first line alternative. ^3^ Available published EUCAST MIC distributions from EUCAST or other laboratories were retrieved and referenced to the right for comparison (range used when few isolates reported). ^4^ Data sets on which these are based are partly truncated at 0.016 mg/L. ^5^ MIC_50_/MIC_90_ were 0.03/0.125 mg/L for serogroup A and AD, which comprise 949 + 177 = 1126 (84%) of the 1334 isolates, while MIC_50_/MIC_90_ were ≤0.015/0.06 mg/L for the remaining 16% belonging to serogroup D in that study [42]. ^6^ MIC values are partly from a data set with a lower truncation at ≤0.03 mg/L. These values were stated as 0.03 and affect the following species (*n* of isolates): *C. intermedia* (2), *C. fermentati* (2), *C. guilliermondii* (3) [44]. EUCAST clinical breakpoints for the common species inserted in solid line/dotted line. The MIC_50_ is underlined (species with ≥10 isolates) and the modal MIC is in bold (≥5 isolates). For isolates with ≥10 isolates, the distribution around the modal MIC/MIC_50_ is marked in shaded grey. S/I/R classification in green/orange/red. NA: Not available. The data set includes the MIC values for candidaemia and non-bloodstream isolates as specified in the Supplementary Text S2.

## 7. Conclusions

In this review we suggested recommendations for interpretation of EUCAST MICs for rare yeasts. Because both the available MIC data and the clinical outcome experience were limited, we adopted a principle of caution by comparing the MIC distributions of the rare species to the more common and thus presumably more pathogenic *Candida* species before classification. Moreover, we confirmed our MICs against those in the literature for given species–drug combinations. It is important to consider how to best report the suggested interpretation to the clinicians. Either the MICs and interpretation can be communicated directly to the clinician, or, alternatively, the interpretation can be reported as S (MICs that fall in the “Treat if wild-type” category), as I (MICs that fall in the “Consider use if wild-type and …” category), and R (MICs that fall in the “Consider alternative therapy” category) according to Table 3, with an appropriate comment highlighting that the categorisation is not based on established EUCAST clinical breakpoints and therefore should be taken with some caution. The wording of such a comment could be:

Formal categorising of the susceptibility of the organism is not possible. The MIC and comparison to other species suggest that the agent may be used for treatment when reported “S”; should be restricted to non-severe cases, high dose therapy, or when no better options are available when reported “I”; and should not be used for therapy when reported “R”.

It is our hope that this pragmatic approach may help in choosing an optimal therapeutic agent for invasive infections due to rare yeasts until approved breakpoints are established.

## Data Availability

Data are only available for research upon reasonable request to Statens Serum Institut and within the framework of the Danish data protection legislation.

## Supplementary Materials

The following supporting information can be downloaded at: https://www.mdpi.com/article/10.3390/jof8020141/s1: List S1: A list of current and previous *Candida* and yeast names used in the manuscript. Text S2: Material and methods section detailing specimen types, antifungals, and years for the Danish isolates included in the MIC data in Table 4, Table 5, Table 6 and Table 7. References [69,70] are cited in the Supplementary Materials
