# Supplementary material for: A Pragmatic Approach to Susceptibility Classification of Yeasts without EUCAST Clinical Breakpoints
Source: J Fungi (Basel). 2022 Jan 30;8(2):141. doi: 10.3390/jof8020141 (PMC8877802; doi:10.3390/jof8020141)
Supplement: Supplementary file 1 [file jof-08-00141-s001.zip › Supplementary List S1.pdf]

S1: Supplementary list of various *Candida* and yeast species names.

This is not a full list showing all taxonomic revisions but is only intended as a help to list selected current and previous names of primarily the species included in this manuscript. Current name is stated in bold.

<u>Current and obsolete names for *Candida* species</u>

C. bovina	***Kazachstania bovina***
C. catenulata	***Diutina catenulata***
C. ciferrii	***Trichomonascus ciferrii***
C. eremophila	***Pichia kluyveri*** *(used)*
C. fabianii	***Cyberlindnera fabianii***, *Pichia fabianii*
C. famata	***Debaryomyces hansenii***
C. fermentati	***Meyerozyma caribbica***, *Pichia caribbica*
C. guilliermondii	***Meyerozyma guilliermondii***
C. inconspicua	***Pichia cactophila***, *Torulopsis inconspicua*
C. intermedia	*Blastodendrion intermedius, Cryptococcus intermedius*
C. kefyr	***Kluyveromyces marxianus***
C. krusei	***Pichia kudriavzevii***, *Isatchenkii orientalis*
C. lambica	***Pichia fermentans***
C. lipolytica	***Yarrowia lipolytica***
C. lusitaniae	***Clavispora lusitaniae***
C. magnoliae	*Torulopsis magnoliae, Starmerella magnoliae*
C. norvegensis	***Pichia norvegensis***
C. palmioleophila	**-**
C. pararugosa	***Wickerhamiella pararugosa***
C. pelliculosa	***Wickerhamomyces anomalus***, *Pichia anomala, Hansenula anomala*
C. pulcherrima	***Metschnikowia pulcherrima***
C. rugosa	***Diutina rugosa***
C. thermophila	***Ogataea polymorpha***, *Hansenula polymorpha*
C. utilis	***Cyberlindnera jadinii***, *Pichia jadinii*

Current and former Non-*Candida* yeast species:

Lodderomyces elongisporus	*Saccharomyces elongisporus*
Magnusiomyces clavatus	*Saprochaeta clavata, Geotrichum clavatum*
Magnusiomyces capitatus	*Geotrichum capitatum, Blastoschizomyces capitatus, Dipodascus capitatus, Saprochaeta capitata, Trichosporon capitatum*
Geotrichum candidum	*Galactomyces candidus, Endomyces/Dipodascus/Galactomyces geotrichum*
Cryptococcus albidus	***Naganishia albida***
**(*Barnettozyma salicaria***	*Pichia salicaria*)
Trichosporon dermatis	***Cutaneotrichosporon dermatis***
(*Williopsis saturnus*	***Cyberlindnera saturnus***, *Saccharomyces saturnus*)
Saccharomyces telluris	***Kazachstania telluris***
Exophiala dermatitidis	*Wangiella dermatitidis*

Based on: de Hoog GS, Guarro J, Gené J, Ahmed S, Al-Hatmi AMS, Figueras MJ & Vitale RG (2020) Atlas of Clinical Fungi, 4th edition. Hilversum except for species names in parentheses (not in the Atlas) found at: *https://www.ncbi.nlm.nih.gov/Taxonomy/Browser/wwwtax.cgi* (both accessed 25.11.2021).
