# Supplementary material for: A Pragmatic Approach to Susceptibility Classification of Yeasts without EUCAST Clinical Breakpoints
Source: J Fungi (Basel). 2022 Jan 30;8(2):141. doi: 10.3390/jof8020141 (PMC8877802; doi:10.3390/jof8020141)
Supplement: Supplementary file 1 [file jof-08-00141-s001.zip › Supplementary Text S2.pdf]

S2: Materials and Methods for the MIC data used and Interpretive Approach

Methods for strain selection. All Danish blood culture yeast isolates submitted for identification and susceptibility testing according to EUCAST E.Def 7 as part of the Danish fungaemia surveillance 2012-2020 were included (only isolates identified to the species level). For blood culture isolates from this period, MALDI-TOF (Bruker) and ITS sequencing were used as previously described [1,2]. This data set was supplemented with those for non-common yeast isolates from other clinical samples (for years 2016 to 2020 from submitted samples and those referred as culture growth for susceptibility testing). Some referred isolates had already been identified at local departments of clinical microbiology (MALDI-TOF is generally implemented in all Danish Departments of Clinical Microbiology starting before 2016). These isolates were only re-identified upon receipt if the identification was inconsistent with morphology or susceptibility findings.

Due to truncated ranges for anidulafungin early 2012, included isolates are from the end of March 2012-2020. For voriconazole, isolates were from 2017-2020 and for amphotericin B, only isolates from 2015 to 2020 were included (as E-tests were used prior to this) [1].

Susceptibility data from our laboratory from 41 published clinical Danish ITS sequenced rare yeasts isolates (*C. intermedia, C. fermentati, C. palmioleophila, C. guilliermondii* and *C. lusitaniae*) isolates obtained during a previous time period were also included [3].

C. auris, an emerging and important pathogen, was not included for two reasons. First, we have not had any cases of *C. auris* in Denmark in 2012-2020 and therefore our collection was limited to a shared collection of *C. auris* isolates from India [4]. Second and more important, *C. auris* from the different parts of the world belong to different clades with a somewhat differential susceptibility and in most cases dominated by clonally related isolates (due to the hospital outbreaks *C. auris* has caused). As per EUCAST SOP 10.2, EUCAST does not accept MIC distributions dominated by isolates from outbreaks for ECOFF setting, as such distributions will be biased towards the outbreak strains and thus not necessarily representative for the broader wild-type of that species (European Committee on Antimicrobial Susceptibility Testing. MIC distributions and epidemiological cut-off value (ECOFF) setting, EUCAST SOP 10.2, 2021. http://www.eucast.org.). Therefore, we have abstained from including *C. auris* in this publication.

Comparison of the MIC values to published EUCAST MIC data and Interpretation for Susceptibility Categories. Our MIC distributions for the common species were next compared to EUCAST distributions (numbers tested, MIC_{50}/MIC_{90} values adapted from the rationale documents for the individual drugs) and

confirmed to be comparable to EUCAST data [5–9]. For non-common species, EUCAST ECOFFs (or tECOFFs) and EUCAST MIC distribution data were retrieved, where available [5–9]. Recent datasets on susceptibility data from other centers using the EUCAST method was found and included for comparison [10–24]. Rare yeast may grow more slowly than common *Candida* species. For some studies, the incubation period was 48 hours and allowed minimal growth was +0.1 OD -which may influence MIC values [10,25].

Finally, treatment recommendations from the ECMM/ISHAM/ASM 2021 Rare Yeast Guideline (based on clinical experience and various susceptibility data) were inserted for the relevant species to give an estimate of known clinical outcome [26].

In total, 4656 isolates were identified. Of these, 718 were rare yeasts (of which 280 isolates were *C. dubliniensis*). Only species with >1 isolate or available references for EUCAST MIC data were displayed in tables 4-7. However, it should be noted that MIC ranges for species with few isolates or few reference MICs should be confirmed with more MIC data when possible. For the most common species, our MICs were nicely comparable to EUCAST MIC distributions used for determination of ECOFFs and CBPs (Tables 4-7). Overall our MIC_{50}s were similar to or one dilution step lower/higher than EUCAST distributions (if allowing a similar truncated lower MIC of 0.016 mg/L for part of the EUCAST voriconazole and fluconazole dataset).

References:

1. Astvad, K.M.T.; Johansen, H.K.; Røder, B.L.; Rosenvinge, F.S.; Knudsen, J.D.; Lemming, L.; Schønheyder, H.C.; Hare, R.K.; Kristensen, L.; Nielsen, L.; et al. Update from a 12-Year Nationwide Fungemia Surveillance: Increasing Intrinsic and Acquired Resistance Causes Concern. *J. Clin. Microbiol.* **2018**, *56*, e01564-17, doi:10.1128/JCM.01564-17.
2. Risum, M.; Astvad, K.; Johansen, H.K.; Schønheyder, H.C.; Rosenvinge, F.; Knudsen, J.D.; Hare, R.K.; Datcu, R.; Røder, B.L.; Antsupova, V.S.; et al. Update 2016-2018 of the Nationwide Danish Fungaemia Surveillance Study: Epidemiologic Changes in a 15-Year Perspective. *J. fungi (Basel, Switzerland)* **2021**, *7*, 491, doi:10.3390/jof7060491.
3. Jensen, R.H.; Arendrup, M.C. Candida palmioleophila: characterization of a previously overlooked pathogen and its unique susceptibility profile in comparison with five related species. *J. Clin. Microbiol.* **2011**, *49*, 549–56, doi:10.1128/JCM.02071-10.
4. Helleberg, M.; Jørgensen, K.M.; Hare, R.K.; Datcu, R.; Chowdhary, A.; Arendrup, M.C. Rezafungin In Vitro Activity against Contemporary Nordic Clinical Candida Isolates and Candida auris Determined by the EUCAST Reference Method. *Antimicrob. Agents Chemother.* **2020**, *64*, doi:10.1128/AAC.02438-19.
5. The European Committee on Antimicrobial Susceptibility Testing. Overview of antifungal ECOFFs and clinical breakpoints for yeasts, moulds and dermatophytes using the EUCAST E.Def 7.3, E.Def 9.3 and E.Def 11.0 procedures. Version 2, 2020. https://www.eucast.org.

6. European Committee on Antimicrobial Susceptibility Testing. Fluconazole: Rationale for the clinical breakpoints, version 3.0, 2020. http://www.eucast.org.

7. European Committee on Antimicrobial Susceptibility Testing. Amphotericin B: Rationale for the clinical breakpoints, version 2.0, 2020 http://www.eucast.org.

8. European Committee on Antimicrobial Susceptibility Testing. Voriconazole: Rationale for the clinical breakpoints, version 4.0 2020. http://www.eucast.org.

9. European Committee on Antimicrobial Susceptibility Testing. Anidulafungin: Rationale for the clinical breakpoints, version 3.0, 2020. http://www.eucast.org.

10. Stavrou, A.A.; Pérez-Hansen, A.; Lackner, M.; Lass-Flörl, C.; Boekhout, T. Elevated minimum inhibitory concentrations to antifungal drugs prevail in 14 rare species of candidemia-causing Saccharomycotina yeasts. *Med. Mycol.* **2020**, *58*, 1–9, doi:10.1093/mmy/myaa005.

11. Fernández-Ruiz, M.; Guinea, J.; Puig-Asensio, M.; Zaragoza, Ó.; Almirante, B.; Cuenca-Estrella, M.; Aguado, J.M. on behalf of CANDIPOP Project and GEIH-GEMICOMED (SEIMC) and REIPI. Fungemia due to rare opportunistic yeasts: data from a population-based surveillance in Spain. *Med. Mycol.* **2017**, *55*, 125–136, doi:10.1093/mmy/myw055.

12. Esposto, M.C.; Prigitano, A.; Lo Cascio, G.; Ossi, C.; Grancini, A.; Cavanna, C.; Lallitto, F.; Tejada, M.; Bandettini, R.; Mularoni, A.; et al. Yeast-like filamentous fungi: Molecular identification and in vitro susceptibility study. *Med. Mycol.* **2019**, *57*, 909–913, doi:10.1093/mmy/myy133.

13. Bretagne, S.; Renaudat, C.; Desnos-Ollivier, M.; Sitbon, K.; Lortholary, O.; Dromer, F. Predisposing factors and outcome of uncommon yeast species-related fungaemia based on an exhaustive surveillance programme (2002–14). *J. Antimicrob. Chemother.* **2017**, *72*, 1784–1793, doi:10.1093/jac/dkx045.

14. Álvarez-Uría, A.; Muñoz, P.; Vena, A.; Guinea, J.; Marcos-Zambrano, L.J.; Escribano, P.; Sánchez-Carrillo, C.; Bouza, E.; Valerio, M.; Cruz, A.F.; et al. Fungaemia caused by rare yeasts: Incidence, clinical characteristics and outcome over 10 years. *J. Antimicrob. Chemother.* **2018**, *73*, 823–825, doi:10.1093/jac/dkx436.

15. Taverna, C.G.; Córdoba, S.; Murisengo, O.A.; Vivot, W.; Davel, G.; Bosco-Borgeat, M.E. Molecular identification, genotyping, and antifungal susceptibility testing of clinically relevant Trichosporon species from Argentina. *Med. Mycol.* **2014**, *52*, 356–366, doi:10.1093/mmy/myt029.

16. Díaz-García, J.; Alcalá, L.; Martín-Rabadán, P.; Mesquida, A.; Sánchez-Carrillo, C.; Reigadas, E.; Muñoz, P.; Escribano, P.; Guinea, J. Susceptibility of uncommon Candida species to systemic antifungals by the EUCAST methodology. *Med. Mycol.* **2020**, *58*, 848–851, doi:10.1093/mmy/myz121.

17. Delma, F.Z.; Al-Hatmi, A.M.S.; Buil, J.B.; van der Lee, H.; Tehupeiory-Kooreman, M.; de Hoog, G.S.; Meletiadis, J.; Verweij, P.E. Comparison of MIC Test Strip and Sensititre YeastOne with the CLSI and EUCAST Broth Microdilution Reference Methods for In Vitro Antifungal Susceptibility Testing of Cryptococcus neoformans. *Antimicrob. Agents Chemother.* **2020**, *64*, 1–7, doi:10.1128/AAC.02261-19.

18. Arabatzis, M.; Abel, P.; Kanellopoulou, M.; Adamou, D.; Alexandrou-Athanasoulis, H.; Stathi, A.; Platsouka, E.; Milioni, A.; Pangalis, A.; Velegraki, A. Sequence-based identification, genotyping and EUCAST antifungal susceptibilities of Trichosporon clinical isolates from Greece. *Clin. Microbiol. Infect.* **2014**, *20*, 777–783, doi:10.1111/1469-0691.12501.

19. Guinea, J.; Zaragoza, Ó.; Escribano, P.; Martín-Mazuelos, E.; Pemán, J.; Sánchez-Reus, F.; Cuenca-Estrella, M. Molecular Identification and Antifungal Susceptibility of Yeast Isolates Causing Fungemia Collected in a Population-Based Study in Spain in 2010 and 2011. *Antimicrob. Agents Chemother.* **2014**, *58*, 1529–1537, doi:10.1128/AAC.02155-13.

20. Gomez-Lopez, A.; Alastruey-Izquierdo, A.; Rodriguez, D.; Almirante, B.; Pahissa, A.; Rodriguez-Tudela, J.L.; Cuenca-Estrella, M.; Fridkin, S.; Hajjeh, R.; Park, B.; et al. Prevalence and susceptibility profile of Candida metapsilosis and Candida orthopsilosis: Results from population-based surveillance of candidemia in Spain. *Antimicrob. Agents Chemother.* **2008**, *52*, 1506–1509, doi:10.1128/AAC.01595-07.

21. Lovero, G.; Borghi, E.; Balbino, S.; Cirasola, D.; De Giglio, O.; Perdoni, F.; Caggiano, G.; Morace, G.; Montagna, M.T. Molecular identification and echinocandin susceptibility of candida parapsilosis complex bloodstream isolates in Italy, 2007-2014. *PLoS One* **2016**, *11*, doi:10.1371/JOURNAL.PONE.0150218.

22. Cendejas-Bueno, E.; Gomez-Lopez, A.; Mellado, E.; Rodriguez-Tudela, J.L.; Cuenca-Estrella, M. Identification of pathogenic rare yeast species in clinical samples: Comparison between phenotypical and molecular methods. *J. Clin. Microbiol.* **2010**, *48*, 1895–1899, doi:10.1128/JCM.00336-10.

23. Beyer, R.; Spettel, K.; Zeller, I.; Lass-Flörl, C.; Achleitner, D.; Krause, R.; Apfalter, P.; Buzina, W.; Strauss, J.; Gregori, C.; et al. Antifungal susceptibility of yeast bloodstream isolates collected during a 10-year period in Austria. *Mycoses* **2019**, 357–367, doi:10.1111/myc.12892.

24. Desnos-Ollivier, M.; Lortholary, O.; Bretagne, S.; Dromer, F. Azole Susceptibility Profiles of More than 9,000 Clinical Yeast Isolates Belonging to 40 Common and Rare Species. *Antimicrob. Agents Chemother.* **2021**, *65*, 1–10, doi:10.1128/AAC.02615-20.

25. Pérez-Hansen, A.; Lass-Flörl, C.; Lackner, M.; Aigner, M.; Alastruey-Izquierdo, A.; Arikan-Akdagli, S.; Bader, O.; Becker, K.; Boekhout, T.; Buzina, W.; et al. Antifungal susceptibility profiles of rare ascomycetous yeasts. *J. Antimicrob. Chemother.* **2019**, *74*, 2649–2656, doi:10.1093/jac/dkz231.

26. Chen, S.C.-A.; Perfect, J.; Colombo, A.L.; Cornely, O.A.; Groll, A.H.; Seidel, D.; Albus, K.; de Almedia, J.N.; Garcia-Effron, G.; Gilroy, N.; et al. Global guideline for the diagnosis and management of rare yeast infections: an initiative of the ECMM in cooperation with ISHAM and ASM. *Lancet Infect. Dis.* **2021**, *3099*, 1–12, doi:10.1016/s1473-3099(21)00203-6.
